# Logarithmically transformed lactate-to-hemoglobin ratio demonstrates a threshold effect on 28-day mortality in sepsis: Analysis of the MIMIC-IV database

**DOI:** 10.1371/journal.pone.0342994

**Published:** 2026-02-26

**Authors:** Huang Chen, Da Ke, Xiuqin Zheng, Haiyang Song, Shirong Lin

**Affiliations:** 1 Shengli Clinical Medical College of Fujian Medical University, Fuzhou, China; 2 Department of Emergency, Fujian Provincial Hospital, Fuzhou, China; 3 Fuzhou University Affiliated Provincial Hospital, Fuzhou, China; 4 Fujian Provincial Key Laboratory of Emergency Medicine, Fuzhou, China; Children's National Hospital, George Washington University, UNITED STATES OF AMERICA

## Abstract

**Objective:**

To investigate the association between logarithmically transformed lactate-to-hemoglobin ratio (Ln_LHR) and 28-day mortality in sepsis patients, addressing the critical need for reliable prognostic biomarkers in this high-mortality condition.

**Methods:**

This retrospective cohort study analyzed 20725 adult sepsis patients from the MIMIC-IV database (2008–2019). The primary exposure was Ln_LHR calculated from measurements within 24 hours of ICU admission, while the primary outcome was 28-day all-cause mortality. Covariates included demographics, physiological parameters, severity scores, and interventions. We employed multivariable logistic regression and restricted cubic splines to identify potential non-linear relationships.Finally,mediation analysis was used to assess the factors affecting sepsis mortality in Ln_LHR.

**Results:**

Elevated Ln_LHR was independently associated with increased 28-day mortality after comprehensive adjustment (OR:1.43, 95% CI:1.35–1.52). We identified a significant threshold effect at Ln_LHR: −0.625, above which mortality risk increased dramatically (OR:3.812, 95% CI:3.131–4.642). Subgroup analyses revealed the predictive efficacy of Ln_LHR exhibited significant variation across various factors, including age, the utilisation of norepinephrine, and the severity score. Mediation analysis revealed that minimum temperature, as a potential mechanism linking Ln_LHR to 28-day mortality, accounted for 9.2% of the total association.

**Conclusion:**

Ln_LHR may represent a promising, readily available prognostic biomarker for sepsis mortality risk stratification, with a clinically meaningful threshold effect. This composite marker integrates critical pathophysiological information and may enhance risk assessment and guide clinical decision-making in sepsis management.

## Introduction

Sepsis represents a global health crisis with substantial mortality rates. In 2017, global sepsis incidence reached 48.9 million cases with 11 million deaths, accounting for approximately 20% of all global deaths [1]. In China, the standardized incidence was 328.25 per 100,000 population in 2019, with an increasing trend [2]. The 28-day mortality rate among sepsis patients remains alarmingly high (30–40%), rising to 50–60% in those with septic shock [3]. Despite substantial advances in early recognition and management [4; 5], identifying reliable prognostic biomarkers for mortality risk stratification remains challenging.

The lactate-to-hemoglobin ratio (LHR), particularly its natural logarithmic form (Ln_LHR), represents a novel composite biomarker integrating critical pathophysiological information. Elevated lactate levels reflect tissue hypoperfusion and cellular metabolic dysfunction, while decreased hemoglobin compromises oxygen-carrying capacity – both fundamental mechanisms in sepsis pathophysiology [6; 7]. Recent evidence suggests that the combination of high lactate and low hemoglobin synergistically exacerbates tissue hypoxia in sepsis patients, creating a vicious cycle strongly associated with adverse outcomes [4; 8]. Recently，a study demonstrated the predictive value of lactate-to-hematocrit ratio for 30-day mortality in sepsis patients, highlighting the potential utility of composite markers in prognostic assessment [9].

This retrospective cohort study using the MIMIC-IV database aimed to investigate the association between Ln_LHR and 28-day mortality in sepsis patients, addressing several knowledge gaps in existing literature. Our investigation uniquely explores the potential non-linear relationship and threshold effects of Ln_LHR on mortality, evaluates its predictive performance across various patient subgroups, and examines possible mediating mechanisms. By evaluating Ln_LHR as a readily available, cost-effective prognostic tool, this study provides clinicians with a practical approach to identify high-risk sepsis patients and potentially inform individualized treatment strategies.

## Methods

### Study population

**Fig 1:**
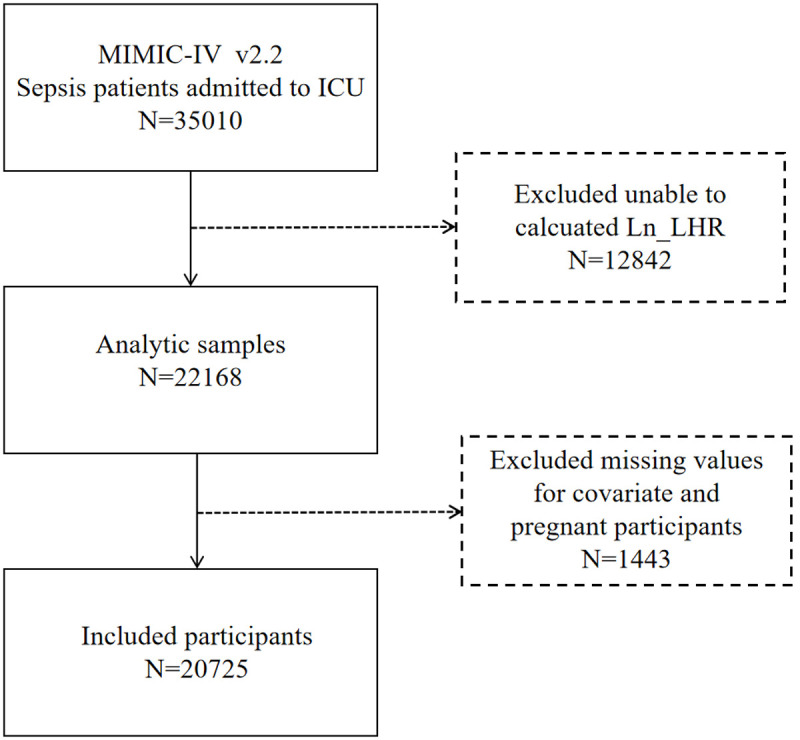
The flow diagram of the study participants.

This retrospective cohort study utilized the MIMIC-IV database (version 2.2), analyzing data from 2008−2019 with 28-day follow-up after ICU admission [10]. The initial cohort comprised 35,010 adult patients (aged 18 years and over) who met the Sepsis-3 criteria [11] (i.e., suspected infection with an acute SOFA score change of ≥2 [6]). These patients were recruited from two major healthcare centres, namely, Massachusetts General Hospital and Beth Israel Deaconess Medical Center. Sepsis diagnosis followed the 2016 international consensus definition. Patients were included if their ICU stay exceeded 24 hours with at least one measurement of both lactate and hemoglobin.We excluded cases with 12,842 missing lactate and hemoglobin data and 1,443 lacking complete covariate information or identified as pregnant. Following the application of the aforementioned exclusions, the final analysis encompassed 20,725 participants who satisfied all the study’s criteria (Fig 1 and S1 Table). All patients were followed from ICU admission until death, discharge, or 28 days, whichever occurred first.

### Variables

The primary exposure was the natural logarithm of the lactate-to-hemoglobin ratio (Ln_LHR), which was calculated using the maximum lactate value(mmol/L) and the minimum hemoglobin(g/dL) ratio within 24 hours of admission to the ICU(Ln_LHR = ln(lactate(mmol/L)/hemoglobin(g/dL))). Logarithmic transformation was selected to reduce outlier influence and approximate normality. Ln_LHR was analyzed primarily as a continuous variable, with supplementary analyses using tertiles to evaluate non-linearity. The primary outcome was 28-day all-cause mortality, determined from MIMIC-IV records by two independent researchers blinded to exposure status. Covariates included demographics (age, sex), physiological parameters (minimum pH, minimum platelet count,maximum heart rate,maximum blood urea nitrogen(BUN),maximum white blood cell count(WBC), maximum creatinine, minimum mean arterial pressure(MAP), minimum temperature,Maximum Respiration rate), severity scores (SOFA, SAPS II, minimum GCS, Charlson Comorbidity Index), and interventions (mechanical ventilation, norepinephrine treatment). These were selected based on established sepsis mortality risk factors and biological plausibility. In this study, Structured Query Language (SQL) was used to extract data through Navicat Premium software (Version 16). All the code for statistical analysis variables and severity scores can be found on the GitHub page: https://github.com/MIT-LCP/mimic-code.

### Ethics statement

The first author of this study completed the collaborative institution training programme course and passed the ‘conflict of interest’ and ‘data or sample research only’ examinations (ID: 13270470). And this study was approved by the Institutional Review Boards of Massachusetts Institute of Technology and Beth Israel Deaconess Medical Center. The requirement for informed consent was waived as MIMIC-IV contains de-identified data and the study was retrospective, in accordance with the Declaration of Helsinki. Data processing adhered to institutional protection policies, with results presented only in aggregate form. The study complies with STROBE guidelines and international ethical standards for medical research, with all personal identifiers fully anonymized before analysis to ensure patient privacy protection.

### Statistical analysis

Continuous variables were presented as mean ± standard deviation or median (interquartile range), and categorical variables as frequencies or percentages. Between-group comparisons used χ² tests, Student’s t-tests, or Mann-Whitney U tests as appropriate. We investigated the association between Ln_LHR and 28-day mortality through progressive multivariable logistic regression models: unadjusted (Model 1), adjusted for gender and age(Model 2), and fully adjusted for all Table 1 covariates (Model 3). This approach examined the robustness of Ln_LHR effect estimates under different confounding control scenarios.

**Table 1:** Baseline characteristics of participants in MIMIC(N = 20725).

Characteristics	Survivors (n = 16758)	Non-survivors (n = 3967)	p
Ln_LHR	−1.5 (−2.0, −1.1)	−1.1 (−1.7, −0.4)	< 0.001
GENDER, n (%)			< 0.001
Male	10072 (60.1)	2220 (56)	
Female	6686 (39.9)	1747 (44)	
Age	65.1 ± 15.8	69.6 ± 15.1	< 0.001
Minimum PH	7.32 ± 0.1	7.25 ± 0.1	< 0.001
Minimum GCS	11.8 ± 3.8	9.6 ± 4.7	< 0.001
Minimum Platelets(*10^9/L)	182.6 ± 105.8	169.7 ± 116.1	< 0.001
Maximum WBC(*10^9/L)	14.2 (10.4, 19.1)	15.6 (10.5, 21.7)	< 0.001
Maximum BUN(mg/dL)	22.0 (15.0, 36.0)	36.0 (22.0, 56.0)	< 0.001
Maximum Creatinine(mg/dL)	1.1 (0.8, 1.7)	1.7 (1.1, 2.8)	< 0.001
Maximum Heart rate(beats/min)	106.4 ± 20.4	114.9 ± 23.6	< 0.001
Minimum MAP(mmHg)	56.7 ± 12.9	50.2 ± 15.8	< 0.001
Maximum Respiration rate(breaths/min)	28.6 ± 6.7	31.1 ± 7.2	< 0.001
Minimum Temperature(℃)	36.2 ± 0.8	36.0 ± 1.1	< 0.001
Mechanical Ventilation, n (%)			< 0.001
No	7181 (42.9)	1310 (33)	
yes	9577 (57.1)	2657 (67)	
Norepinephrine, n (%)			< 0.001
No	11821 (70.5)	1257 (31.7)	
Yes	4937 (29.5)	2710 (68.3)	
SOFA	6.6 ± 3.5	10.4 ± 4.3	< 0.001
SAPS II	39.5 ± 13.4	54.2 ± 15.9	< 0.001
Charlson Comorbidity Index	5.7 ± 2.9	7.2 ± 2.9	< 0.001

Data are presented as unweighted number (weighted percentage) for categorical variables and mean (SE) for continuous variables.Ln_LHR:logarithmically transformed lactate-to-hemoglobin ratio; GCS:Glasgow Coma Scale; WBC:white blood cell; BUN:blood urea nitrogen; MAP:mean arterial pressure.

To address potential non-linearity, we employed generalized additive modeling with smooth curve fitting, calculating inflection points when non-linearity was detected and constructing two-piecewise logistic regression models. We conducted subgroup analyses using stratified models, with continuous stratification variables converted to categories using clinical cut points or tertiles. Finally, the mediation analysis explored the mediating role of body temperature in the association between Ln_LHR and sepsis mortality. All analyses were performed using R software (version 4.3.2) and Free Statistics software(version 2.2.0) [12; 13], with two-sided P values <0.05 considered statistically significant.

## Result

### Baseline characteristics

Baseline characteristic analysis revealed significant differences between the 28-day survival group and the mortality group (Table 1). The proportion of male patients in the study population was significantly higher than that of female patients, with a 28-day all-cause mortality rate of 19.1%. A comparison of the deceased group with the surviving patients revealed that the former had a higher mean age, and significantly higher mean values for maximum heart rate, SAPS II score, Charlson Comorbidity Index, minimum GCS score, and SOFA score (p < 0.05). However, the minimum values of mean blood pressure and body temperature were significantly lower (p < 0.05). Furthermore, patients who received mechanical ventilation and norepinephrine therapy exhibited higher mortality rates (p < 0.001). With regard to laboratory indicators, the Ln_LHR at admission was found to be significantly higher in the mortality group than in the survival group (p < 0.001). The minimum pH values and platelet counts were found to be significantly lower in the mortality group (p < 0.05), while the maximum values of white blood cell counts, blood urea nitrogen, and creatinine levels were significantly higher (p < 0.05).

### Association between Ln_LHR and 28-day mortality in sepsis

The present study utilised multivariable logistic regression analysis to investigate the association between Ln_LHR and 28-day mortality in sepsis. As demonstrated in Table 2, Ln_LHR exhibited a positive correlation with 28-day mortality in sepsis. In all models, including the unadjusted model (Model 1, OR:2.33, 95% CI:2.22–2.44), p < 0.001), the partially adjusted model (Model 2, OR:2.4, 95% CI:2.29–2.52), p < 0.001), and the fully adjusted model (Model 3, OR:1.43, 95% CI:1.35–1.52), p < 0.001), a consistent positive correlation was observed. The findings suggest that in the fully adjusted model, a one-unit increase in Ln_LHR corresponds to a 43% increase in the 28-day mortality rate from sepsis. Furthermore, in comparison with individuals in the lowest tertile (Q1), those in the highest tertile (Q3) of Ln_LHR exhibited a 35% higher mortality rate (Model 3, OR:1.35, 95% CI(1.21–1.5), p < 0.001).

**Table 2:** Multivariate logistic regression analysis of risk factors for death in patients within 28 days.

Characteristics	Model 1 OR (95%CI)	Model 2 OR (95%CI)	Model 3 OR (95%CI)
Ln_LHR	2.33 (2.22 ~ 2.44)	2.4 (2.29 ~ 2.52)	1.43 (1.35 ~ 1.52)
Categories			
Q1	1(Ref)	1(Ref)	1(Ref)
Q2	1.23 (1.11 ~ 1.35)	1.23 (1.11 ~ 1.35)	0.96 (0.86 ~ 1.07)
Q3	2.96 (2.71 ~ 3.23)	3.05 (2.79 ~ 3.33)	1.35 (1.21 ~ 1.5)
p for trend	< 0.001	< 0.001	< 0.001

Ln_LHR:logarithmically transformed lactate-to-hemoglobin ratio; Model 1:unadjusted; Model 2:adjusted for gender and age; Model 3:fully adjusted for all Table 1 covariates.

**Fig 2:**
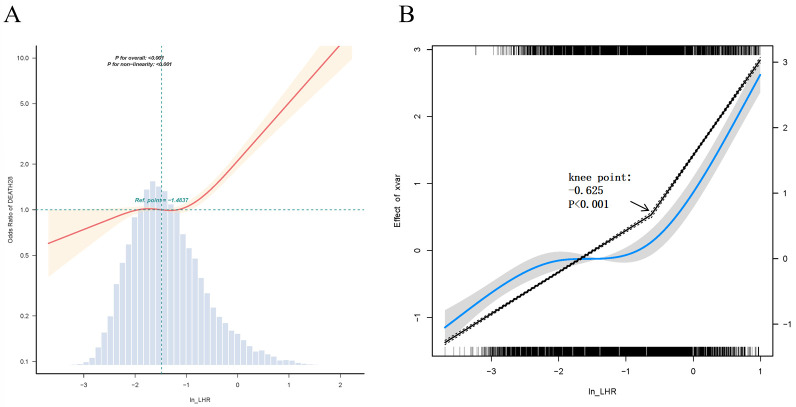
The nonlinear association between mortality and Ln_LHR.

The application of a smooth curve fit to the fully adjusted model yielded further insights into the nonlinear relationship between Ln_LHR and 28-day mortality (Fig 2A). Concurrently, we employed likelihood ratio tests and bootstrap resampling methods to identify the inflection point. The result identified an inflection point at Ln_LHR:-0.625(−0.692,-0.558) (Fig 2B). The data reveal that, below the threshold, each unit increase in the variable was associated with an 64.6% higher risk (OR:1.646, 95% CI: 1.518–1.785, P < 0.001), while above the threshold, each unit increase was associated with a 281.2% higher mortality risk (OR:3.812, 95% CI: 3.131–4.642, P < 0.001). The effect magnitude above the threshold was found to be significantly stronger than below.

**Fig 3:**
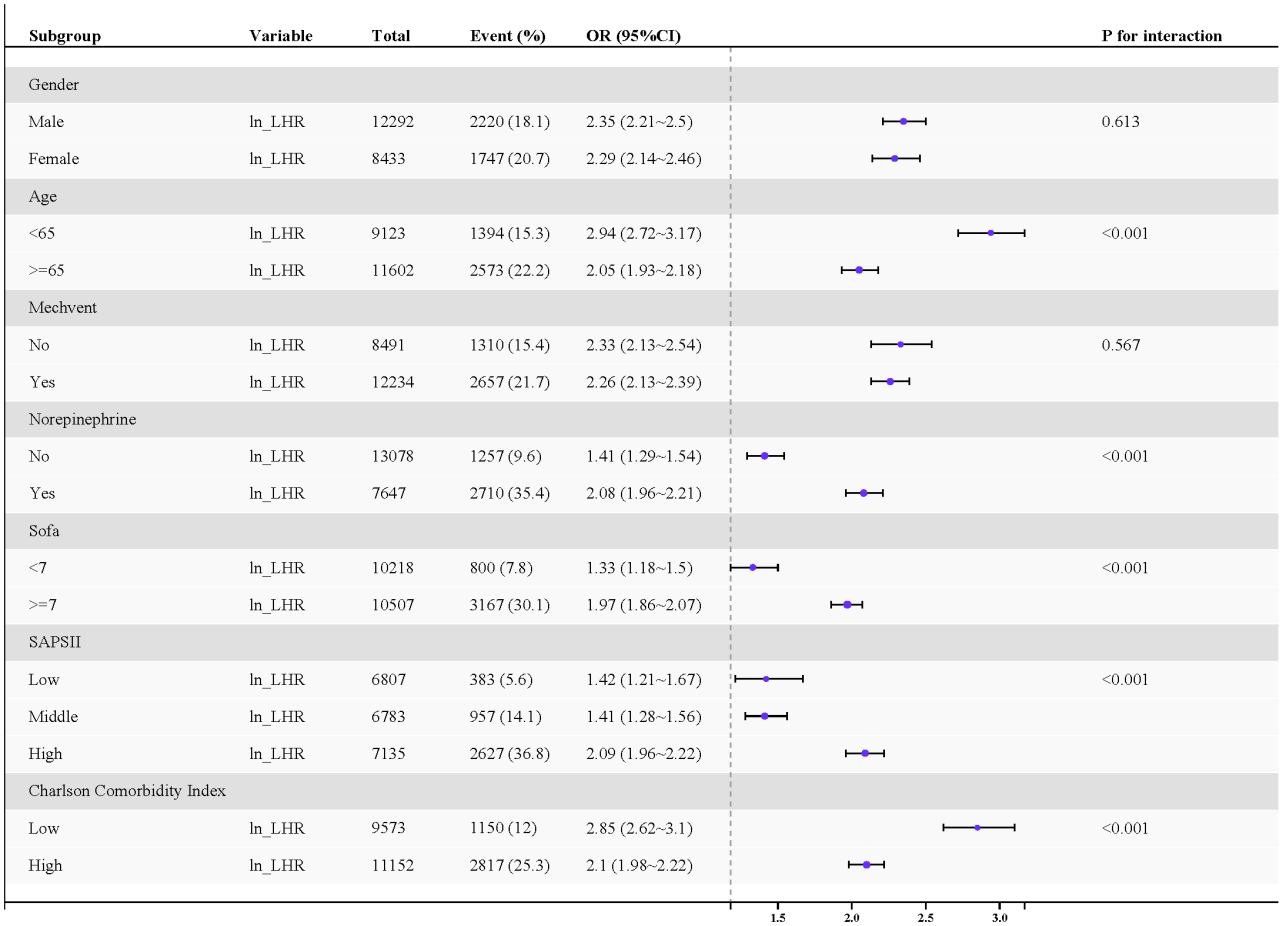
Forest plot for subgroup analysis of the relationship between mortality and Ln_LHR.

A stratified analysis was conducted, incorporating factors such as age, gender, use of norepinephrine, use of a ventilator, SAPS II, Charlson Comorbidity Index, and SOFA score (Fig 3). The forest plot (Fig 3) demonstrates an absence of statistically significant interaction between Ln_LHR and gender or ventilator use (interaction p-values of 0.613 and 0.567, respectively). However, the results of the subgroup analysis indicate a robust association between Ln_LHR and 28-day mortality from sepsis across different subgroups(All P < 0.05).

### Mediation analysis

**Fig 4:**
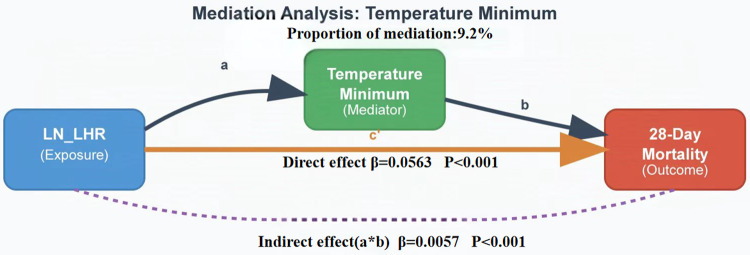
the mediating effect of temperature on the Ln_LHR related mortality.

Causal mediation analysis examining minimum body temperature as a potential mechanism linking Ln_LHR with 28-day mortality revealed that the total effect of Ln_LHR on mortality was 0.0619 (95% CI: 0.0502-0.0748, P < 0.001) (Fig 4). This effect primarily consisted of a direct effect (0.0563, 95% CI: 0.0449-0.069, P < 0.001), with temperature minimum mediating only 9.2% of the total association. The mediation effect, while statistically significant (0.0056, 95% CI: 0.0042-0.0071, P < 0.001), was modest in magnitude compared to the direct pathyway.

## Discussion

In this comprehensive analysis involving sepsis patients from the MIMIC-IV database, we investigated the association between Ln_LHR and 28-day mortality. We found that increased Ln_LHR was significantly associated with higher 28-day mortality in sepsis patients, with each unit increase conferring 35% higher odds of death after comprehensive adjustment (OR:1.43, 95% CI:1.35–1.52). Notably, we identified a clinically significant threshold effect at Ln_LHR: −0.625, above which mortality risk increased dramatically (OR:3.812, 95% CI:3.131–4.642), suggesting that Ln_LHR represents a promising prognostic biomarker for risk stratification in sepsis management. And temperature is an intermediary factor between Ln_LHR and mortality.

During sepsis, changes in lactate and hemoglobin levels reflect tissue oxygenation and metabolic disorders, and may also amplify the inflammatory response through a series of reactions [14; 15]. Firstly, elevated lactate levels indicate enhanced glycolytic compensation and mitochondrial dysfunction [16]. Lactate is no longer simply considered a ‘product of anaerobic metabolism’ [17]. For instance, it can activate the HIF-1α/NLRP3 axis, promoting the release of IL-1β by monocytes and exacerbating systemic inflammation [18]. Secondly, low hemoglobin levels impair the ‘oxygen transport network’ in the blood. Studies have shown that hemoglobin undergoes a pathological transformation in sepsis involving oxidative stress-release of free hemoglobin-activation of the TLR4 pathway [19]. Together, these two factors amplify tissue hypoxia, triggering an inflammatory storm and endothelial damage, which can ultimately lead to multi-organ failure. Recent animal experiments have shown that combining a lactate scavenger (dichloroacetate) with a hemoglobin chelator (haptoglobin) can significantly reduce the level of inflammation [20; 21]. Therefore, monitoring the ratio of blood lactate to plasma free hemoglobin dynamically may serve as a new indicator for assessing the intensity of ‘inflammation-oxidation’ coupling, providing a basis for timing precise transfusion and anti-inflammatory therapy.

Our findings on Ln_LHR’s prognostic value in sepsis align with recent evidence while offering methodological innovations. Using the MIMIC-IV database, a study demonstrated that lactate-to-hematocrit ratio predicted 30-day mortality in sepsis patients [9]. Our study extends this work by employing logarithmic transformation to address non-linearity and identifying a precise threshold effect at Ln_LHR: −0.625, enabling more nuanced risk stratification. In contrast, a study explored the lactate-to-albumin ratio (LAR) as an alternative marker, showing superior performance compared to its individual components [22]. This difference in biomarker selection reflects distinct pathophysiological mechanisms: while albumin primarily indicates inflammatory status, hemoglobin directly impacts oxygen-carrying capacity—critical in sepsis where tissue hypoxia predominates [23; 24]. The superior predictive performance of Ln_LHR likely stems from its unique ability to simultaneously capture two key processes: elevated lactate indicating tissue hypoperfusion [23], and reduced hemoglobin reflecting both inflammatory erythrocyte damage and hemodilution from fluid resuscitation. This dual reflection of metabolic dysfunction and compromised oxygen delivery provides a more comprehensive assessment of sepsis severity than single biomarkers, explaining the enhanced discriminatory power observed in our analysis.

The clinical utility of Ln_LHR offers a powerful yet accessible approach to sepsis prognostication. Our identification of a threshold at Ln_LHR: −0.625 provides a clinically actionable parameter that stratifies patients into distinct risk categories, enabling evidence-based triage decisions in critical care settings. Ln_LHR’s advantage stems from its integration of complementary pathophysiological information—lactate reflecting cellular metabolic stress and hemoglobin representing oxygen-carrying capacity—using routinely obtained laboratory values. The logarithmic transformation addresses non-linearity between the ratio and mortality risk, enhancing both statistical validity and clinical interpretability. We propose incorporating Ln_LHR into existing risk-stratification algorithms to guide time-sensitive interventions, including fluid resuscitation strategies and vasopressor initiation. Its simplicity makes it particularly valuable in resource-constrained settings where sophisticated biomarkers may be unavailable. Future research should examine whether serial Ln_LHR measurements provide incremental prognostic information, whether Ln_LHR-guided therapeutic algorithms improve patient outcomes, and whether its predictive accuracy varies across different sepsis endotypes and infection sources—ultimately refining our understanding of sepsis heterogeneity and informing personalized management strategies.This study found that although temperature regulation disorders are a statistically significant mediating pathway, and multiple studies have shown that changes in body temperature may be predictive of sepsis [25; 26; 27], they only explain a small portion of the impact of Ln_LHR on mortality.The predominant mechanisms likely involve other physiological pathways not captured by temperature changes alone, suggesting Ln_LHR affects outcomes through multiple pathophysiological mechanisms rather than primarily through temperature disturbances. Clinically, these results suggest that interventions targeting temperature regulation alone would likely have limited impact on improving outcomes in patients with elevated Ln_LHR, highlighting the need for more comprehensive therapeutic approaches addressing the broader physiological derangements.

Our study demonstrates several methodological strengths enhancing the reliability and clinical relevance of our findings. We utilized a large, well-characterized cohort from the MIMIC-IV database, providing robust statistical power while adjusting for multiple confounders. It should be noted that numerous other confounding factors remain, in addition to those considered in this study. Further research is required to refine the applicability and accuracy of these conclusions in the future. The application of logarithmic transformation to the lactate-to-hemoglobin ratio effectively addressed non-linearity between this biomarker and mortality outcomes, improving model fit and interpretability. The comprehensive adjustment strategy encompassing demographics, vital signs, and laboratory parameters minimized potential confounding.

However,several limitations of our study warrant consideration. First,as a retrospective analysis from a single tertiary care center in the United States, our findings may not fully generalize to other healthcare settings with different patient demographics or clinical practices.In particular, with regard to the diagnostic aspects of the subject, blood culture,the current gold standard for the diagnosis of sepsis,has certain limitations, including lengthy turnaround times, low sensitivity, and the potential for contamination [28]. In future, further research will be conducted among patients diagnosed with sepsis under more stringent criteria. Second, despite comprehensive adjustments, the observational nature of our study precludes definitive causal inferences about the relationship between Ln_LHR and mortality.Third,we could not account for unmeasured confounding factors such as pre-hospital care quality, time to antibiotic administration, or genetic factors that might influence both Ln_LHR values and outcomes. Fourth, our study utilized only baseline Ln_LHR measurements at admission, whereas serial measurements might better capture sepsis dynamics and provide additional prognostic information. Finally, without prospective validation, it remains unclear whether interventions targeting Ln_LHR would improve clinical outcomes. These limitations emphasize the need for external validation in diverse populations before widespread clinical implementation.

## Conclusion

This study showed that Ln_LHR may serve as a promising prognostic indicator for the 28-day mortality risk in patients with sepsis.This new finding is a powerful additional tool for identifying the prognosis of sepsis patients, enabling us to closely monitor high-risk patients and intervene early in treatment.

## Supporting information

S1 Table The datas of participants in MIMIC. (XLSX)
